# Non‐invasive pressure–volume loop analysis in left ventricular load manipulation

**DOI:** 10.1111/cpf.70081

**Published:** 2026-07-13

**Authors:** Karin Pola, Charlotte Burup Kristensen, William D. Watson, Peregrine G. Green, Betty Raman, Stefan Neubauer, Oliver J. Rider, Christian Hassager, Rasmus Møgelvang, Per M. Arvidsson

**Affiliations:** ^1^ Department of Clinical Sciences, Lund, Clinical Physiology Skåne University Hospital Lund, Lund University Lund Sweden; ^2^ Cardiology, Department of Clinical Sciences, Lund Lund University Lund Sweden; ^3^ Department of Cardiology, The Heart Center Copenhagen University Hospital Rigshospitalet Copenhagen Denmark; ^4^ Division of Cardiovascular Medicine University of Cambridge Cambridge UK; ^5^ Oxford Centre for Magnetic Resonance Research University of Oxford Oxford UK; ^6^ Department of Physiology, Anatomy and Genetics University of Oxford Oxford UK; ^7^ Department of Clinical Medicine, Faculty of Health and Medical Sciences Copenhagen University Copenhagen Denmark

**Keywords:** afterload, contractility, hemodynamic monitoring, preload, stroke work, ventricular‐arterial coupling, ventricular efficiency

## Abstract

**Background:**

Clinical monitoring of patients with heart failure or cardiomyopathy is facilitated by detailed assessment of cardiac loading conditions. Specifically, alterations in preload and afterload may unmask pathology through effects on ventricular pressure–volume (PV) relations. Therefore, the aim was to assess non‐invasive PV loops during left ventricular load manipulation in healthy participants and in patients with hypertrophic cardiomyopathy (HCM).

**Methods:**

In total, *n* = 46 participants were studied in paired experiments at baseline and during load manipulation. Controls (*n* = 24) and patients with HCM (*n* = 14) were assessed at baseline and during intravenous infusion of 1.5–2 L isotonic saline, and another group of volunteers (*n* = 8) was assessed at baseline and during infusion of glyceryl trinitrate (GTN). Non‐invasive PV loops were calculated from cardiovascular magnetic resonance (CMR) and concurrent brachial blood‐pressure measurements.

**Results:**

Saline infusion brought about increased end‐diastolic and decreased end‐systolic volumes in controls, but not in HCM. Concurrently, PV loops revealed mechanistic differences, where controls but not HCM exhibited decreased arterial elastance and potential energy during load manipulation. In both groups, ventricular‐arterial coupling (VAC) decreased, and ventricular efficiency and cardiac output increased. Infusion of GTN resulted in decreased ventricular volumes. Contrary to saline infusion, volunteers receiving GTN retained unchanged arterial elastance, VAC, ventricular efficiency and cardiac output.

**Conclusion:**

Non‐invasive PV analysis from CMR detects cardiac response to altered loading conditions in healthy participants and in patients with HCM. Our findings support the use of CMR‐derived PV loops for detailed assessment of cardiac thermodynamic performance in relation to interventions affecting preload and afterload.

**Abbreviations**
BP: blood pressure
bSSFP: balanced steady‐state free precession
CMR: cardiovascular magnetic resonance
*E*_A_
: arterial elastance
*E*_max_
: maximum elastance
GTN: glyceryl trinitrate
HCM: hypertrophic cardiomyopathy
LV: left ventricle
PV loop: pressure–volume loop
STROBE: Strengthening the Reporting of OBservational Studies in Epidemiology
*V*_0_
: volume at 0 ventricular pressure
VAC: ventricular‐arterial coupling

## 1 INTRODUCTION

Current guidelines recommend cardiovascular magnetic resonance (CMR) imaging for assessment of cardiac structure and function in patients with suspected heart failure (McDonagh et al., 2021) or cardiomyopathy (Ommen et al., 2020), but also acknowledge the need for more extensive and patient‐specific measurements of cardiac mechanics and hemodynamics. Pressure–volume (PV) analysis is a valuable tool for evaluation of cardiac function (Sagawa et al., 1977; Suga and Sagawa, 1973; Suga, 1969), which provides information complementary to standard volumetric measurements that may support clinical decision‐making (Ky et al., 2013).

While analysis of left ventricular PV relations (PV loops) has traditionally required invasive measurements of intracavitary pressures, recent technical developments have enabled non‐invasive computation of PV loops (Seemann et al., 2019). The method combines standard CMR images for accurate assessments of cardiac volumes and concordant brachial blood‐pressure measurements to estimate the left ventricular PV relations for a given hemodynamic state. This approach has been tested against invasive PV measurements in experimental animal models (Seemann et al., 2019, 2024) and in humans (Arvidsson et al., 2023). Previous studies have used non‐invasive PV loops to demonstrate hemodynamic changes following myocardial infarction in a porcine model (Berg et al., 2023), cardiac unloading after surgical closure of atrial septal defect (Sjöberg et al., 2024) and during sympathetic stimulation using dobutamine in healthy controls (Sjöberg et al., 2021) and in patients with right ventricular volume overloading (Sjöberg et al., 2022). Furthermore, the method has been applied to study changes in myocardial efficiency following metabolic substrate manipulation in heart failure (Watson et al., 2023).

Unloading the failing heart brings about energetic benefits, leading to improvements in long‐term morbidity and mortality (McMurray et al., 2014). Clinical monitoring of treatment response is facilitated by detailed assessment of cardiac loading conditions (McDonagh et al., 2021; Ommen et al., 2020). Specifically, alterations in preload and afterload may unmask pathology through effects on PV relations in patients with aberrant ventricular filling, such as hypertrophic cardiomyopathy (HCM), although this remains to be evaluated using non‐invasive methods.

The aim of this study was therefore to investigate the ability of non‐invasive PV loops to detect cardiac response to load alteration, by assessments at rest and during manipulation of preload and afterload in healthy hearts and in patients with HCM.

## 2 METHODS

### 2.1 Study design

**Figure 1:**
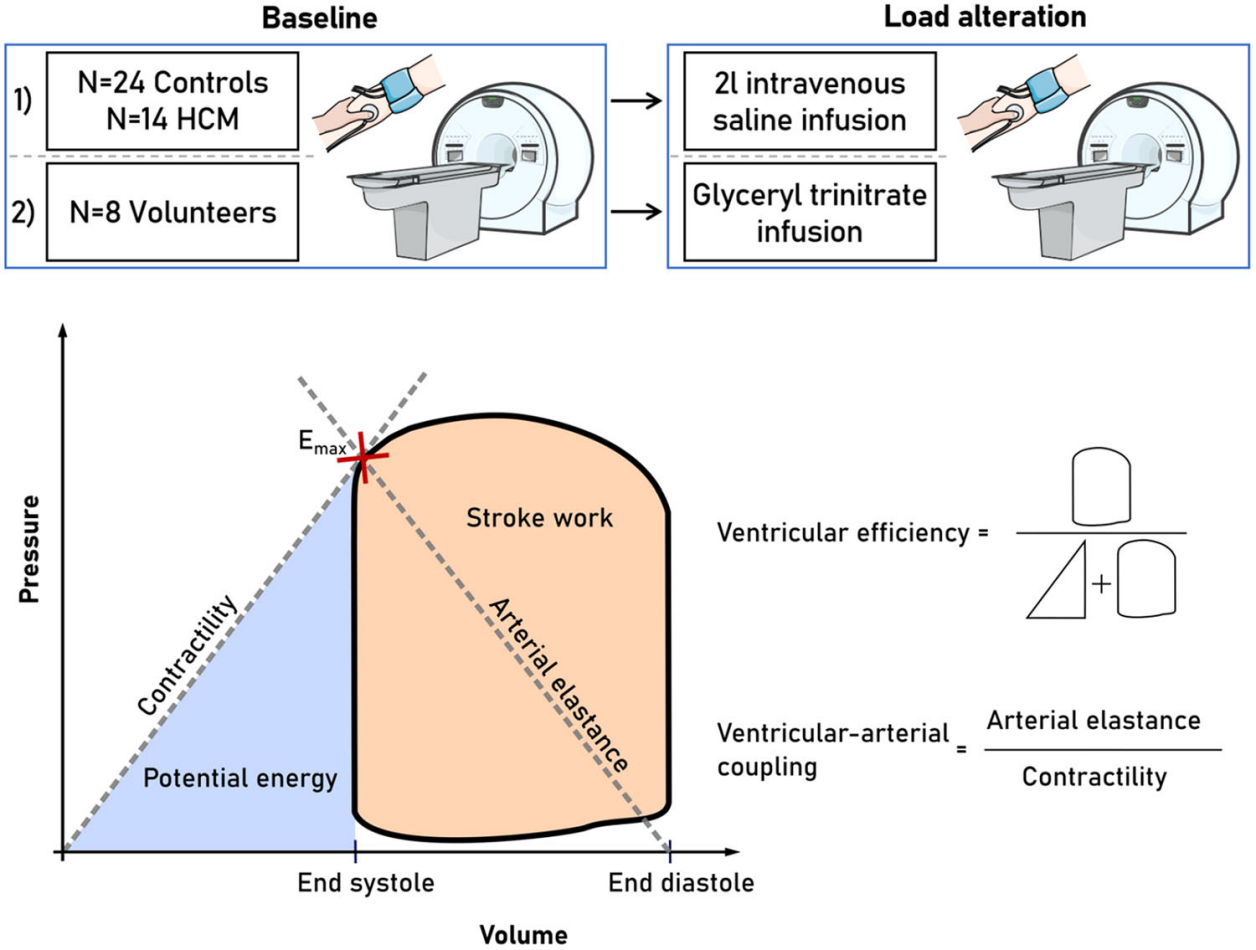
Study overview and PV‐loop parameters. Top: Two sets of experiments were performed to assess the effects of preload and afterload alteration on non‐invasive pressure–volume (PV) loops. (1) Controls and patients with HCM were studied at rest and during infusion of isotonic saline. (2) Volunteers were studied at rest and during infusion of glyceryl trinitrate. Bottom: From the PV‐loops, we computed contractility and arterial elastance as the respective slope of the dashed lines, as well as ventricular‐arterial coupling as the ratio of arterial elastance/contractility, stroke work as the area of the loop, potential energy as the area of the blue triangle, and ventricular efficiency according to the formula in the figure. *E*_max_, maximum elastance; HCM, hypertrophic cardiomyopathy.

This study is comprised of two sets of experiments, summarized in Figure 1, where study participants were examined with paired experiments at baseline and during load manipulation. First, we studied patients with HCM and healthy controls before and after rapid intravenous administration of isotonic saline (experimental details below). This cohort was recruited and studied at Rigshospitalet (Copenhagen, Denmark) and has previously been described in greater detail (Visby et al., 2023). All study participants were recruited from the clinical inflow of scheduled routine echocardiographic examinations, including participants both with and without known cardiovascular disease. Inclusion criteria for both patients and controls were age ≥18 years and sinus rhythm on the day of the examination, and for patients, a confirmed HCM diagnosis. Exclusion criteria for all participants were any contraindication to CMR, heart failure with New York Heart Association (NYHA) class > I, pregnancy or breastfeeding. In addition, exclusion criteria for controls also included any known cardiovascular disease, defined as moderate‐severe valve disease, ischaemic heart disease, HCM, dilated cardiomyopathy, arrhythmogenic right ventricle cardiomyopathy, hypertensive heart disease and Marfan syndrome, as well as any cardiovascular medications. These participants are henceforth referred to as HCM or controls as appropriate.

Second, we studied another cohort at rest and during continuous infusion of glyceryl trinitrate (GTN). These participants were recruited and studied at the John Radcliffe Hospital (Oxford, United Kingdom) as previously described (Watson et al., 2021). To emphasize that this cohort of healthy subjects was not compared to a group of patients, we henceforth refer to them as volunteers. Volunteers were recruited from local oral and written advertisements. The inclusion criterion was age ≥18 years. Exclusion criteria were any history of cardiovascular or metabolic disease, or any contraindications to nitrate‐based medications or to CMR examination.

The present study was approved by the University of Oxford Medical Sciences Interdivisional Research Ethics Committee (R64397/RE001) and the Local ethics committee of the Capital Region of Denmark (H‐16029778) and conducted in accordance with the Declaration of Helsinki. All participants provided written informed consent after receiving written and oral information before study enrolment, and the study was reported in keeping with the Strengthening the Reporting of OBservational Studies in Epidemiology (STROBE) recommendations (Altman et al., 2007).

### 2.2 CMR examination and load manipulation

The cohort with controls and patients with HCM underwent CMR at 1.5 T (Optime MR450W; GE Healthcare), and the cohort with volunteers underwent CMR at 3 T (Tim Trio; Siemens Healthineers). The CMR examinations included acquisition of retrospectively electrocardiogram (ECG)‐gated balanced steady‐state free precession (bSSFP) cine images during end‐expiratory breath‐hold, in long‐axis projections and a short‐axis stack covering the entire left ventricle (LV). Image acquisition parameters are described in greater detail in previous studies (Visby et al., 2023; Watson et al., 2021).

For the saline experiments, a baseline examination was conducted where controls and HCM patients underwent CMR imaging with concurrent brachial blood pressure (BP) measurements. Subsequently, participants exited the scanner and received a rapid infusion of isotonic (0.9%) saline over 30–45 min while resting outside the scanner room, targeting a maximal tolerated dose as determined by the attending physician. Participants were then immediately re‐examined while receiving an additional slower infusion of 0.5 L saline to maintain an increased preload despite extravasation and elimination. The total infusion volume in controls was 2.0 L [2.0–2.0] (median [interquartile range]) and in HCM 1.8 L [1.2–2.0]. BP was measured after completion of the infusion.

For the GTN experiments, volunteers underwent one imaging session with data acquired at baseline and during GTN infusion. The infusion of 1 mg/mL GTN was initiated at a rate of 3 mL/h and titrated at 5‐min intervals to a target of a mean arterial pressure reduction of 15 mmHg, which was maintained during the remainder of the scan. Brachial BP was measured during the acquisition of CMR images at rest and during GTN infusion using an automatic sphygmomanometer (Expression™ MR200 MRI safe monitoring system; MR Devices).

### 2.3 Analysis of non‐invasive PV loops

PV loops were calculated at baseline and during load alteration in each study participant, using the software Segment v.4.1.0.2 (https://medviso.com/segment/). The workflow has been described in greater detail elsewhere (Arvidsson et al., 2023; Seemann et al., 2019). Briefly, LV volumes were defined from manual contouring of endocardial and epicardial borders in end‐diastole and end‐systole, followed by semi‐automatic, time‐resolved contouring of endocardial borders throughout the remaining time phases of the cardiac cycle. Left ventricular pressures were calculated by scaling a time‐varying elastance function to the brachial BP using a previously validated method (Arvidsson et al., 2023; Seemann et al., 2019, 2024) and plotted against the time‐resolved LV volume to produce a PV loop. End‐diastolic pressure was estimated to 7.5 mmHg, and *V*_0_ was set to 0, as in previous studies (Arvidsson et al., 2023; Pola et al., 2024; Seemann et al., 2019). From the PV loops, we then computed left ventricular contractility, arterial elastance, ventricular‐arterial coupling (VAC; *E*_A_/*E*_max_ ratio), stroke work, potential energy and ventricular efficiency (Figure 1).

### 2.4 Statistical analysis

Statistical analysis was conducted in Prism v.10.3.1 (GraphPad Software). Continuous data are presented as median and interquartile range. The Wilcoxon test was used for paired intragroup comparisons, and the unpaired Mann–Whitney *U* test was used for comparisons between controls and HCM. A correlation matrix with Pearson correlations was used for explorative analysis of associations between changes in PV‐loop parameters and changes in volumes and pressures. From the correlation matrix, we identified relevant parameters for further analysis with simple linear regressions and coefficients of determination. A two‐tailed *p*‐value < 0.05 was considered significant. Due to the explorative nature of the study, post hoc Bonferroni correction was then applied to compensate for multiple comparisons regarding PV loop parameters; both uncorrected and corrected p values are given below.

## 3 RESULTS

**Figure 2:**
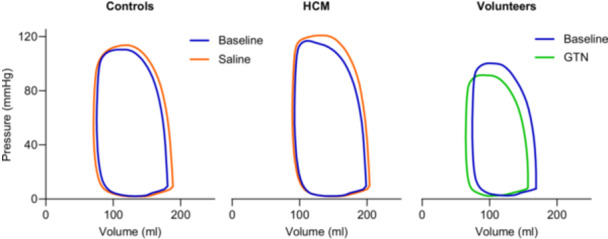
Average pressure–volume loops with alterations in preload and afterload. Each pressure–volume loop is computed from the average values in the respective cohort at a given hemodynamic state. Controls and patients with HCM were examined at baseline and during saline infusion, while another group of volunteers was examined at baseline and during infusion of GTN. GTN, glyceryl trinitrate; HCM, hypertrophic cardiomyopathy.

In total, *n* = 46 participants were enrolled in the present study, of which *n* = 24 were controls, and *n* = 14 were HCM patients receiving saline infusion, and *n* = 8 were volunteers receiving GTN infusion (Figure 1). Each group was analysed using intra‐group comparisons of cardiac hemodynamics and PV loop parameters, comparing values at baseline and during load alteration. Study participant characteristics are summarized in Table 1. Of note, patients with HCM were significantly older (55 vs. 35 years, *p*= 0.001) and had a larger proportion of males (86% vs. 46%, *p*= 0.02) compared to the group of controls. In the group of patients with HCM, *n* = 5 were obstructive, and *n* = 9 were non‐obstructive. Figure 2 shows average PV loops for each group at baseline and during load alteration.

**Table 1:** Study participant characteristics.

	Controls *n* = 24	HCM *n* = 14	*p* Controls versus HCM	Volunteers *n* = 8
Sex, F/M	13/11	2/12	**0.02**	5/3
Age	35 [27–46]	55 [43–61]	**0.001**	36 [26–44]
Height, cm	177 [171–186]	176 [171–183]	0.7	168 [167–178]
Weight, kg	76 [65–83]	80 [76–86]	0.2	59 [58–70]
BSA, m^2^	1.89 [1.77–2.07]	1.99 [1.94–2.04]	0.3	1.65 [1.62–1.87]

*Note*: Data are presented as median [interquartile range]. *p* values below significance level shown in bold.

Abbreviations: BSA, body surface area; F, female; HCM, hypertrophic cardiomyopathy; M, male.

### 3.1 Saline infusion

Saline infusion brought about changes in cardiac pumping physiology in controls and in patients with HCM, as analysed with paired comparisons within each group (Table 2). In controls, end‐diastolic volume increased (*p* = 0.0006) and end‐systolic volume decreased (*p* = 0.01). While a similar trend was seen in HCM, the differences were not statistically significant on the group level. In both controls and patients with HCM, there was an increase in heart rate (*p* = 0.0003 and *p* = 0.004), stroke volume (*p* = 0.00001 and *p* = 0.009), ejection fraction (*p* = 0.00003 and *p* = 0.02) and cardiac output (*p* = 0.00001 and *p* = 0.002). Systolic BP was essentially unchanged in controls but increased slightly in HCM patients. Diastolic BP remained unchanged in both groups.

**Table 2:** Hemodynamic response to saline infusion in controls and HCM.

	Controls			Change after load alteration	HCM			Change after load alteration
	Baseline	Post saline	*p*		Baseline	Post saline	*p*	
Heart rate, bpm	55 [53–65]	66 [59–71]	**0.0003**	6 [1–17]	51 [48–57]	57 [50–62]	**0.004**	4 [1–10]
Systolic BP, mmHg	121 [111–130]	121 [114–136]	**0.04**	5 [−2 to 12]	121 [113–143]	130 [117–156]	0.056	5 [−1 to 16]
Diastolic BP, mmHg	74 [66–80]	73 [69–80]	1.0	0 [−6 to 6]	74 [63–92]	74 [66–90]	0.3	2 [−3 to 7]
End‐diastolic volume, mL	171 [152–200]	176 [165–207]	**0.0006**	8 [3–14]	208 [168–230]	217 [179–232]	0.08	4 [−2 to 14]
End‐systolic volume, mL	69 [63–85]	65 [58–72]	**0.01**	−2 [−11 to 1]	90 [75–105]	89 [70–97]	0.1	−5 [−8 to −1]
Stroke volume, mL	101 [91–121]	116 [107–135]	**0.00001**	12 [3–18]	113 [87–123]	125 [99–134]	**0.009**	7 [3–17]
LV EF, %	59 [55–63]	64 [61–66]	**0.00003**	4 [1–7]	55 [51–58]	58 [55–61]	**0.02**	3 [1–6]
Cardiac output, L/min	6.0 [5.1–7.1]	8.1 [6.7–9.1]	**0.00001**	1.8 [0.2–3.0]	5.9 [4.8–6.2]	7.2 [4.8–8.0]	**0.002**	1.1 [0.4–1.7]

*Note*: Data are presented as median [interquartile range] with *p* values of comparisons within each group. *p* values below significance level shown in bold.

Abbreviations: BP, blood pressure; EF, ejection fraction; HCM, hypertrophic cardiomyopathy; LV, left ventricular.

**Figure 3:**
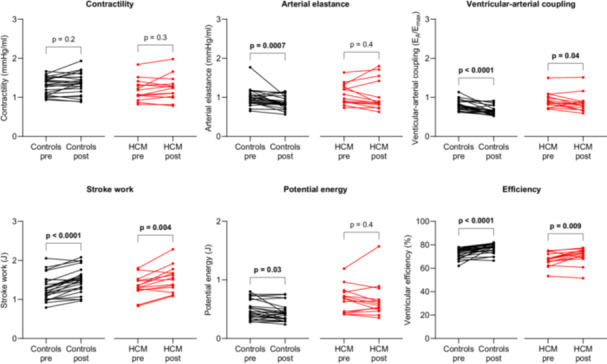
Effects of saline infusion on pressure–volume loop parameters. Left ventricular contractility, arterial elastance, ventricular‐arterial coupling, stroke work, potential energy and ventricular efficiency, measured in controls (black) and patients with hypertrophic cardiomyopathy (HCM) (red) at baseline and during saline infusion. The Wilcoxon test was used for paired intragroup comparisons, and *p* values show paired comparisons within each group.

PV loops at baseline and during saline infusion revealed different mechanistic responses to load alteration in the two groups (Table 3 and Figure 3), where controls exhibited decreased arterial elastance and potential energy during load manipulation, which was not seen in HCM. In both groups, stroke work and ventricular efficiency increased, VAC decreased and contractility remained unchanged. When applying a Bonferroni correction with a *p* value threshold of <0.0083, calculated as 0.05/6, the results remained significant for controls in arterial elastance, VAC, stroke work and ventricular efficiency. In patients with HCM, only the results for stroke work remained significant after Bonferroni correction.

**Table 3:** Effects of saline infusion on pressure–volume loop parameters in controls and HCM.

	Controls			Change after load alteration	HCM			Change after load alteration
	Baseline	Post saline	*p*		Baseline	Post saline	*p*	
Contractility, mmHg/mL	1.3 [1.1–1.5]	1.4 [1.2–1.5]	0.2	0.02 [−0.04 to 0.2]	1.1 [1.0–1.4]	1.3 [1.0–1.4]	0.3	0.05 [−0.05–0.1]
Arterial elastance, mmHg/mL	0.97 [0.88–1.1]	0.83 [0.79–1.0]	**0.0007**	−0.09 [−0.2 to −0.03]	1.0 [0.87–1.3]	0.86 [0.79–1.5]	0.4	−0.05 [−0.2 to 0.1]
Ventricular‐arterial coupling, *E*_A_/*E*_max_	0.75 [0.65–0.87]	0.63 [0.55–0.70]	**<0.0001**	−0.12 [−0.16 to −0.02]	0.88 [0.79–1.0]	0.79 [0.67–0.89]	**0.04**	−0.10 [−0.17 to 0.008]
Stroke work, J	1.3 [1.0–1.5]	1.5 [1.3–1.6]	**<0.0001**	0.2 [0.07–0.3]	1.3 [1.2–1.5]	1.5 [1.3–1.7]	**0.004**	0.2 [0.05–0.3]
Potential energy, J	0.46 [0.36–0.63]	0.42 [0.33–0.50]	**0.03**	−0.04 [−0.09 to 0.02]	0.68 [0.44–0.80]	0.58 [0.48–0.71]	0.4	−0.04 [−0.1 to 0.07]
Efficiency, %	73 [69–77]	79 [75–81]	**<0.0001**	4 [3–5]	68 [64–73]	73 [69–76]	**0.009**	4 [1–7]

*Note*: Data are presented as median [interquartile range] with *p* values of comparisons within each group. *p* values below significance level shown in bold.

Abbreviation: HCM, hypertrophic cardiomyopathy.

#### 3.1.1 Associations with hemodynamic response

**Figure 4:**
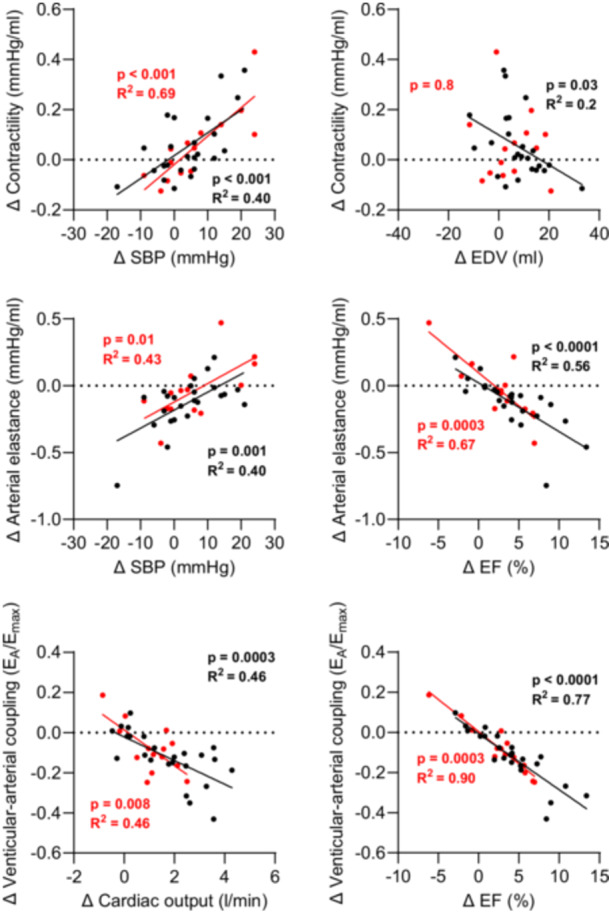
Associations between changes in contractility, arterial elastance and ventricular‐arterial coupling (*E*_a_/*E*_max_ ratio) and volumetric or pressure response to saline infusion in controls (black) and patients with HCM (red). *E*_a_, arterial elastance; *E*_max_, maximum elastance; EDV, end‐diastolic volume; EF, ejection fraction; HCM, hypertrophic cardiomyopathy; SBP, systolic blood pressure.

Figure 4 shows associations between hemodynamic response to saline infusion and changes in PV‐loop parameters related to end‐systolic PV relationships, including contractility, arterial elastance and VAC. Changes in contractility were associated with changes in systolic BP in both groups. In controls, but not in HCM, changes in contractility were also associated with changes in end‐diastolic volume.

Furthermore, arterial elastance was associated with changes in systolic BP and with changes in ejection fraction in both groups. Both groups also exhibited associations between changes in VAC and changes in cardiac output and, as expected, changes in ejection fraction.

**Figure 5:**
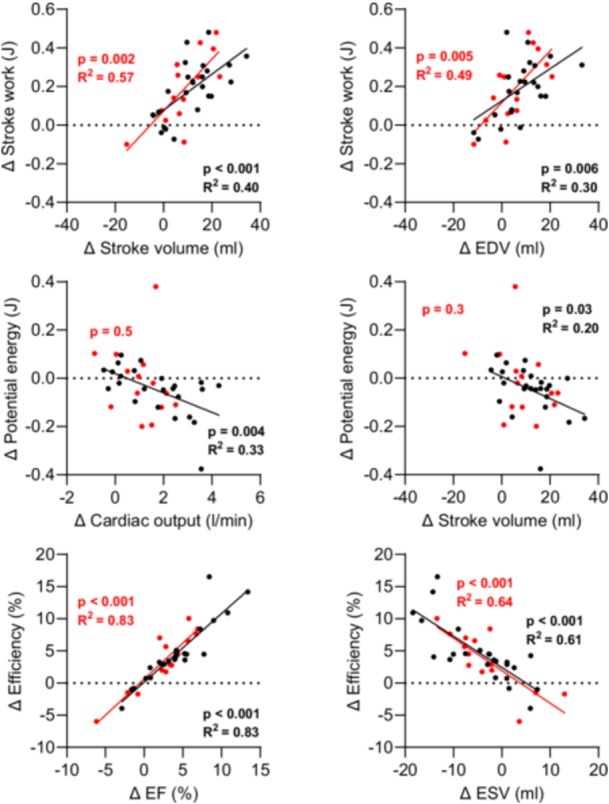
Associations between changes in stroke work, potential energy and ventricular efficiency and volumetric or pressure response to saline infusion in controls (black) and patients with HCM (red). EDV, end‐diastolic volume; EF, ejection fraction; ESV, end‐systolic volume; HCM, hypertrophic cardiomyopathy.

Figure 5 shows associations between the hemodynamic response to saline infusion and changes in PV‐loop parameters related to ventricular work, including stroke work, potential energy and ventricular efficiency.

In both groups, changes in stroke work were associated with changes in stroke volume and changes in end‐diastolic volume. In controls, but not in HCM, changes in potential energy were associated with changes in cardiac output and with changes in stroke volume. As expected, changes in ventricular efficiency were associated with changes in ejection fraction and changes in end‐systolic volume.

### 3.2 GTN infusion

GTN infusion resulted in increased heart rate and ejection fraction, as well as decreased systolic and diastolic BPs, end‐diastolic and end‐systolic volumes and stroke volume, while cardiac output remained unchanged (Table 4).

**Table 4:** Hemodynamic response to GTN infusion in volunteers.

	Volunteers			Change after load alteration
	Baseline	During GTN	*p*	
Heart rate, bpm	69 [60–80]	79 [75–87]	**0.008**	8 [3–15]
Systolic BP, mmHg	111 [101–118]	101 [96–104]	**0.008**	−8 [−13 to −6]
Diastolic BP, mmHg	66 [58–72]	54 [46–58]	**0.008**	−14 [−16 to −11]
End‐diastolic volume, mL	154 [127–172]	138 [118–147]	**0.008**	−16 [−21 to −9]
End‐systolic volume, mL	67 [55–72]	58 [50–60]	**0.008**	−11 [−13 to −5]
Stroke volume, mL	85 [72–101]	80 [68–88]	**0.04**	−5 [−10 to −1]
LV EF, %	57 [54–58]	59 [56–59]	**0.02**	2 [0–3]
Cardiac output, L/min	5.9 [5.1–6.2]	6.1 [5.1–6.8]	0.6	0.0 [−0.3 to 1.0]

*Note*: Data are presented as median [interquartile range]. *p* values below significance level in bold.

Abbreviations: BP, blood pressure; EF, ejection fraction; GTN, glyceryl trinitrate; LV, left ventricular.

**Figure 6:**
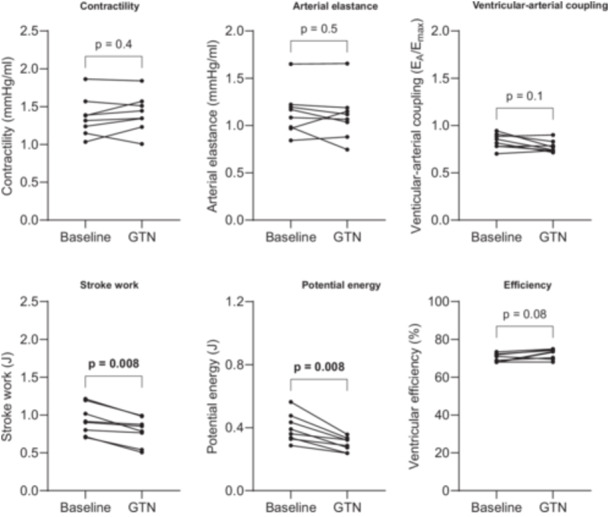
Effects of glyceryl trinitrate (GTN) infusion on pressure–volume loop parameters. Left ventricular contractility, arterial elastance, ventricular‐arterial coupling, stroke work, potential energy and ventricular efficiency, measured in volunteers at baseline and during GTN infusion. The Wilcoxon test was used for paired intragroup comparisons.

GTN infusion in volunteers resulted in decreased stroke work and potential energy (Table 5 and Figure 6), indicating an unloading of the ventricle (*p* < 0.01 for both), together with a trend toward increased ventricular efficiency (*p* = 0.08). No statistically significant changes were seen for contractility, arterial elastance or VAC. When applying Bonferroni correction with a *p* value threshold of <0.0083, the results for stroke work and potential energy remained significant.

**Table 5:** Effects of GTN infusion on pressure–volume loop parameters in volunteers.

	Volunteers			Change after load alteration
	Baseline	During GTN	*p*	
Contractility, mmHg/mL	1.3 [1.2–1.5]	1.4 [1.3–1.6]	0.4	0.0 [−0.1 to 0.2]
Arterial elastance, mmHg/ml	1.1 [0.97–1.2]	1.1 [0.92–1.2]	0.5	0.0 [−0.12 to 0.03]
Ventricular‐arterial coupling, *E*_A_/*E*_max_	0.84 [0.78–0.90]	0.76 [0.73–0.82]	0.1	−0.06 [−0.11 to 0.01]
Stroke work, J	0.91 [0.74–1.2]	0.82 [0.60–0.96]	**0.008**	−0.18 [−0.23 to −0.04]
Potential energy, J	0.38 [0.33–0.47]	0.30 [0.25–0.33]	**0.008**	−0.11 [−0.14 to −0.04]
Efficiency, %	70 [68–72]	73 [70–75]	0.08	2 [0–5]

*Note*: Data are presented as median [interquartile range]. *p* values below significance level in bold.

Abbreviation: GTN, glyceryl trinitrate.

#### 3.2.1 Associations with hemodynamic response

**Figure 7:**
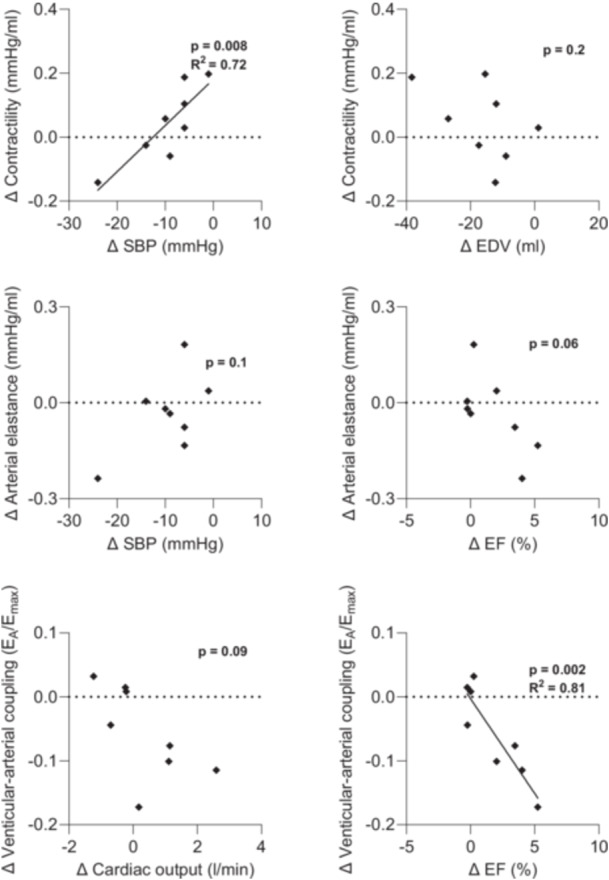
Associations between changes in contractility, arterial elastance and ventricular‐arterial coupling (*E*_a_/*E*_max_ ratio) and volumetric or pressure response to glyceryl trinitrate infusion in volunteers. *E*_a_, arterial elastance; *E*_max_, maximum elastance; EDV, end‐diastolic volume; EF, ejection fraction; SBP, systolic blood pressure.

Figure 7 shows associations between the hemodynamic response to GTN infusion and changes in PV‐loop parameters related to end‐systolic PV relationships. Changes in contractility were strongly associated with changes in systolic BP (*R* ^2^ = 0.72), but not with changes in end‐diastolic volume. While visual trends could be observed between changes in arterial elastance and changes in systolic BP and changes in ejection fraction, neither reached statistical significance. VAC, which takes into account both contractility and arterial elastance, displayed a non‐significant trend towards decreasing with increasing cardiac output, and as expected, was strongly associated with changes in ejection fraction (*R* ^2^ = 0.81).

**Figure 8:**
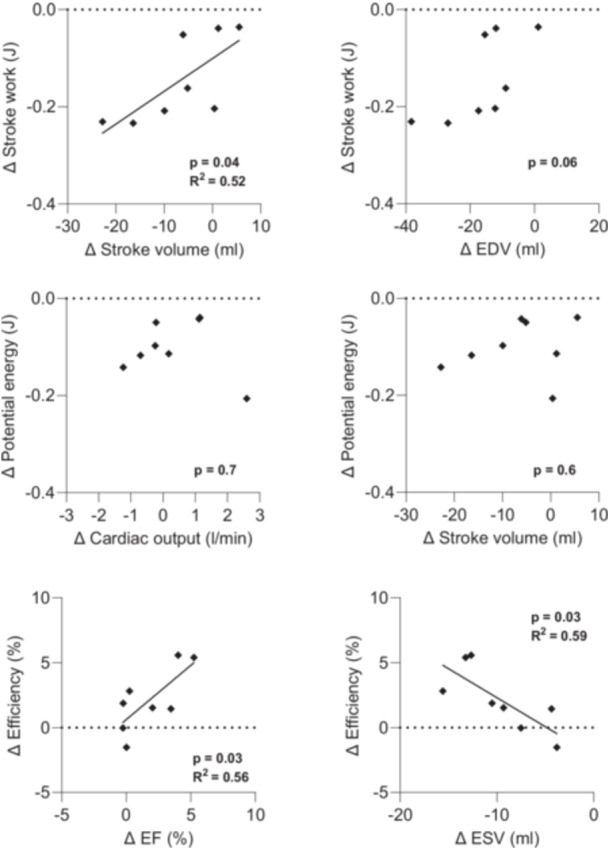
Associations between changes in stroke work, potential energy and ventricular efficiency and volumetric or pressure response to glyceryl trinitrate infusion in volunteers. EDV, end‐diastolic volume; EF, ejection fraction; ESV, end‐systolic volume.

Figure 8 shows associations between hemodynamic response to GTN infusion and changes in PV‐loop parameters related to stroke work. Similar to saline infusion in controls, the results for GTN infusion showed associations between changes in stroke work and changes in stroke volume, as well as between changes in ventricular efficiency and changes in ejection fraction and changes in end‐systolic volume. Contrary to saline infusion, GTN infusion did not result in associations between changes in stroke work and changes in end‐diastolic volumes, nor between changes in potential energy and changes in cardiac output or changes in stroke volume.

## 4 DISCUSSION

This study demonstrates that non‐invasive PV loops can detect physiological responses to altered loading conditions, showing distinct differences between healthy participants and patients with HCM. Saline infusion produced clear changes in controls, with less pronounced responses in HCM, while GTN infusion produced the expected unloading pattern without major alterations in elastance or VAC. Alterations in PV‐loop parameters were associated with hemodynamic response to load modification, confirming CMR‐derived PV loops capture load‐dependent behaviour in both healthy and diseased hearts. Importantly, these responses were detected despite coarse load interventions, heterogeneous patient characteristics and non‐simultaneous PV acquisition, underscoring the intrinsic sensitivity of the method.

### 4.1 Physiological response to altered load

All three groups of study participants exhibited faster heart rates during load manipulation compared to baseline. Increased heart rate was expected in the cohort receiving GTN, since the vasodilator decreases BP and thereby is prone to stimulate sympathetic upregulation, resulting in a faster heart rate. In the cohort receiving saline infusion, increased heart rate was seen in combination with unaltered or slightly increased blood pressure in both controls and patients with HCM. Here, the heart‐rate response is likely a sign of the baroreceptor reflex combined with the psychological stress of receiving 2 L of fluid, whereas the relatively unaffected BP indicates that a significant amount of the administered fluid is distributed into the venous system or extravascular compartments.

In all groups, hemodynamic adaptations to load alteration stimulated changes in PV‐loop parameters, as demonstrated by association analyses. Nonetheless, a coefficient of determination ranging from 0.20 to 0.90 indicates that volumetric and pressure alterations are not the sole explanation behind changes in PV loops, and that dynamic PV‐loop analysis may provide information beyond conventional imaging‐based analysis of cardiac function.

In both controls and patients with HCM, stroke work increased after saline infusion, while contractility remained unchanged. In 1996, Pak et al. studied invasive PV loops in patients with HCM recorded during preload variations using a balloon‐catheter obstruction of the inferior vena cava (Pak et al., 1996). Patients with HCM displayed a flat diastolic PV relation curve, which shifted downward on reduction of preload, meaning a net pressure decline at similar chamber volumes, which was not seen in controls, or in patients with LV hypertrophy secondary to chronic hypertension, or in patients with idiopathic dilated cardiomyopathy. The authors conclude that patients with HCM exhibit unique PV relations during ventricular filling, which may impact the interpretation of ventricular stiffness. Patients with HCM often exhibit a restrictive physiology, and so assumptions built into the non‐invasive PV‐loop model may not be entirely adequate in this patient cohort.

### 4.2 Clinical implications

Clinical management of heart failure aims to relieve the chronic volume loading imposed on the heart. Non‐invasive PV‐loops may provide detailed and patient‐specific assessment of cardiac loading conditions complementary to standard diagnostic instruments. Previous studies have demonstrated that non‐invasive PV‐loop analysis may offer clinically valuable information and guide risk stratification beyond standard hemodynamic parameters in patients with heart failure (Arvidsson et al., 2024) and in patients with myocardial infarction (Lav et al., 2025; Nordlund et al., 2024). The findings in our study demonstrate that non‐invasive PV loops capture subtle load‐dependent changes within subjects, seen in both healthy controls and in HCM, suggesting a role for monitoring cardiac thermodynamic response to therapeutic interventions. Since the HCM cohort in this study included different phenotypes and heterogeneous medical regimes, the PV observations should not be interpreted as physiological characteristics of HCM. Rather, they demonstrate that non‐invasive PV‐loop analysis can detect meaningful hemodynamic responses even under comparatively coarse interventions. The methodological sensitivity observed under these broad conditions therefore justifies future studies with more stringently controlled PV interventions, aimed at defining potential clinical utility in selected patient cohorts.

This non‐invasive approach to PV evaluation entails less risk and discomfort for patients compared to invasive tools. Moreover, volume measurements on CMR provide outstanding accuracy and precision compared to invasive catheters. Combining gold‐standard volumetry from CMR with a validated model for estimating LV pressures, non‐invasive PV loops thus have potential to match or even outperform invasive measurements of PV relations, as previously suggested (Arvidsson et al., 2023; Edlund et al., 2022). This approach provides safe, precise and widely accessible surrogate markers of outcome in clinical studies, and facilitates monitoring of treatment response in patients whose risk/benefit ratio does not motivate invasive procedures. Furthermore, previously acquired datasets can be retrospectively analysed for large‐scale cohort studies of cardiac thermodynamics and its association with relevant outcomes (Arvidsson et al., 2024).

### 4.3 Limitations

The blood‐pressure measurements in the study participants receiving saline were obtained in conjunction with, but not simultaneously with, the acquisition of short‐axis cine images used for volume analysis. Moreover, the experimental protocol used for the saline infusions meant the study participants experienced a variable but significant bladder distension, a non‐trivial physiological and psychological stressor. The stress associated with urinary urgency will have affected the balance between parasympathetic and sympathetic activation, the relative magnitude of which is unknown. Furthermore, as the PV‐loop model requires a user‐estimated end‐diastolic pressure, and assumes the volume‐axis intercept ‘*V*_0_’ is set to 0, severe deviations in preload, end‐diastolic pressures and ventricular stiffness may be masked, possibly slightly overestimating stroke work and efficiency, and underestimating contractility (Seemann et al., 2019).

Few patients were included in this study, and we did not examine distinct disease subgroups. The GTN cohort was also modest in size, which limits the precision of effect estimates, although the observed responses were consistent with the expected physiological consequences of nitrate‐induced unloading. These findings nevertheless indicate that non‐invasive PV‐loop analysis can detect load‐dependent hemodynamic changes even in small cohorts.

## 5 CONCLUSION

Non‐invasive PV analysis from CMR detects cardiac response to altered loading conditions in healthy participants and in patients with HCM. Our findings support the use of CMR‐derived PV loops for detailed assessment of cardiac thermodynamic performance in relation to interventions affecting preload and afterload.

## CONFLICT OF INTEREST STATEMENT

The authors declare no conflict of interest.

## Supporting information

Supporting File 1

## Data Availability

Anonymized and exported data underlying this article may be shared on reasonable request to the corresponding author. Imaging data are not available for sharing.
